# Enhanced antitumor activity of a novel, oral, helper epitope-containing WT1 protein vaccine in a model of murine leukemia

**DOI:** 10.1186/s12885-023-10547-5

**Published:** 2023-02-20

**Authors:** Hikaru Minagawa, Yoshiko Hashii, Hiroko Nakajima, Fumihiro Fujiki, Soyoko Morimoto, Jun Nakata, Toshiro Shirakawa, Takane Katayama, Akihiro Tsuboi, Keiichi Ozono

**Affiliations:** 1 Department of Pediatrics, Osaka University Graduate School of Medicine, Suita, Japan; 2 Department of Pediatrics, Osaka International Cancer Institute, Osaka, Japan; 3 Department of Cancer Immunology, Osaka University Graduate School of Medicine, Suita, Japan; 4 Department of Cancer Stem Cell Biology, Osaka University Graduate School of Medicine, Suita, Japan; 5 Department of Clinical Laboratory and Biomedical Sciences, Osaka University Graduate School of Medicine, Suita, Japan; 6 Kobe University Graduate School of Science, Technology and Innovation JP, Kobe, Japan; 7 Division of Integrated Life Science, Graduate School of Biostudies, Kyoto University, Kyoto, Japan; 8 Department of Cancer Immunotherapy, Osaka University Graduate School of Medicine, Suita, Japan

**Keywords:** WT1 protein, Immunotherapy, Oral vaccine, CD4^+^ T cell help, WT1-specific cytotoxic T cells, Effector memory T cells, Tumor-infiltrating lymphocytes, Intestinal immunity, Murine leukemia

## Abstract

**Background:**

A Wilms’ tumor 1 (WT1) oral vaccine, *Bifidobacterium longum* (*B. longum*) 420, in which the bacterium is used as a vector for WT1 protein, triggers immune responses through cellular immunity consisting of cytotoxic T lymphocytes (CTLs) and other immunocompetent cells (e.g., helper T cells). We developed a novel, oral, helper epitope-containing WT1 protein vaccine (*B. longum* 2656) to examine whether or not *B. longum* 420/2656 combination further accelerates the CD4^+^ T cell help-enhanced antitumor activity in a model of murine leukemia.

**Methods:**

C1498-murine WT1—a genetically-engineered, murine leukemia cell line to express murine *WT1*—was used as tumor cell. Female C57BL/6 J mice were allocated to the *B. longum* 420, 2656, and 420/2656 combination groups. The day of subcutaneous inoculation of tumor cells was considered as day 0, and successful engraftment was verified on day 7. The oral administration of the vaccine by gavage was initiated on day 8. Tumor volume, the frequency and phenotypes of WT1-specific CTLs in CD8^+^ T cells in peripheral blood (PB) and tumor-infiltrating lymphocytes (TILs), as well as the proportion of interferon-gamma (INF-γ)-producing CD3^+^CD4^+^ T cells pulsed with WT1_35–52_ peptide in splenocytes and TILs were determined.

**Results:**

Tumor volume was significantly smaller (*p* < 0.01) in the *B. longum* 420/2656 combination group than in the *B. longum* 420 group on day 24. WT1-specific CTL frequency in CD8^+^ T cells in PB was significantly greater in the *B. longum* 420/2656 combination group than in the *B. longum* 420 group at weeks 4 (*p* < 0.05) and 6 (*p* < 0.01). The proportion of WT1-specific, effector memory CTLs in PB increased significantly in the *B. longum* 420/2656 combination group than in the *B. longum* 420 group at weeks 4 and 6 (*p* < 0.05 each). WT1-specific CTL frequency in intratumoral CD8^+^ T cells and the proportion of IFN-γ-producing CD3^+^CD4^+^ T cells in intratumoral CD4^+^ T cells increased significantly (*p* < 0.05 each) in the *B. longum* 420/2656 combination group than in the 420 group.

**Conclusions:**

*B. longum* 420/2656 combination further accelerated antitumor activity that relies on WT1-specific CTLs in the tumor compared with *B. longum* 420.

## Background

To date, a diversity of therapeutic modalities for malignant hematologic tumors (e.g., surgery, radiotherapy, chemotherapy, and stem cell transplantation) have been developed and have extensively been applied to the clinical settings. Outstanding recent advances in caner immunotherapy have allowed the clinical application of immune checkpoint inhibitors [1] and chimeric antigen receptor T cell therapy [2]. Nevertheless, the development of a new therapeutic modality is required because the efficacy thereof for different types of malignant tumors is not yet sufficient.

Wilms’ tumor gene 1 (*WT1*), which was originally discovered as a protooncogene, is currently known to be expressed in many tumors [3, 4], and the product of the gene—WT1 protein—is a promising tumor-associated antigen (TAA) that was ranked 1st among 75 cancer antigens [5] and is considered as stem cell antigen [6]. WT1 protein was identified a novel TAA because WT1 peptide-specific cytotoxic T lymphocytes (CTLs) had cytotoxic activity on WT1-expressing target cells [7]. These findings motivated the development of an intradermal WT1 peptide vaccine [8, 9]. Although its sufficient efficacy was demonstrated, a novel oral WT1 protein vaccine is required for cancer patients (especially, pediatric patients), which is devoid of pain- and scarring-causing adjuvant Montanide™ and instead uses *Bifidobacterium longum* (*B. longum*)—a normal intestinal bacterium that is a probiotic with established safety acting as an adjuvant [10] and has low human leukocyte antigen (HLA) restriction because of protein nature [11].

CD4^+^ T cell help is critically important for the clonal expansion of CTLs and their differentiation into effector and memory CTLs [12], for the induction of effector and memory CTL responses [12], and for the infiltration of CTLs into tumors [13]. Previously, an oral vaccine, *B. longum* 420 was developed, in which the intestinal bacterial strain was used as a vector for WT1 protein [14]; the vaccine exhibited anticancer activity in mice. However, *B. longum* 420 had a drawback of not containing a major histocompatibility complex (MHC) class II-restricted epitope important in the induction of WT1-specific Th1 response [15]. To overcome the drawback, we newly developed *B. longum* 2656 that contains the CD4^+^ cell help-inducing, MHC class II-restricted helper epitope (WT1 amino acid sequences 35–52 WAPVLDFAPPGASAYGSL).

The objective of the present study was to examine whether or not *B. longum* 420/2656 combination further accelerates the CD4^+^ T cell help-enhanced antitumor activity in a model of murine leukemia.

## Materials and methods

### Animals and study design

A total of 150 female C57BL/6 J (H-2D^b^) mice, purchased from CLEA Japan, Inc. (Tokyo, Japan), were used for experiments (8–42 animals/experiment) at 6–8 weeks of age. Animals were included in the study when the tumor was measurable on day 7 after subcutaneous inoculation. Animals were excluded from the study when tumor engraftment was unsuccessful on day 7 after subcutaneous inoculation.

### Study materials

1) *B. longum* wild type—the negative control group: *B. longum* 105-A. 2) *B. longum* 420—the positive control group: a partial murine-WT1 amino acid residue 117–419 via galacto-N-biose/lacto-N-biose I-binding protein (GLBP), a membrane protein of wild-type *B. longum* [14]. 3) *B. longum* 2656 displaying a partial murine WT1 protein—an amino acid sequence 26–56 (GLPVSGARQWAPVLDFAPPGASAYGSLGGPA) containing an MHC class II-restricted helper epitope, an amino acid sequence 35–52 (WAPVLDFAPPGASAYGSL) capable of inducing CD4^+^ T cell help. *B. longum* wild type was cultured anaerobically in the Gifu Anaerobic Medium (GAM) broth (Nissui, Tokyo, Japan) at 37 °C, and *B. longum* 420 and 2656 in the GAM broth with 15 μg/mL spectinomycin (Sigma-Aldrich, St. Louis, MO) at 37 °C. These materials were washed with phosphate-buffered saline (PBS) and were then suspended to gain the final cell density of 2 × 10^9^ colony-forming units (CFUs)/100 μL.

### Cells

To prepare C1498-murine WT1 (C1498-mWT1), a murine leukemia cell line C1498 purchased from ATCC (Rockville, MD) was genetically transduced with CMV promotor-driven murine WT1 17AA(+) KTS(+) isoform full-length cDNA that had been inserted into the pcDNA3.1(+) mammalian expression vector (Invitrogen, Tokyo, Japan).

### Study procedure to examine tumor volume

The number of tumor cells was increased by 2 passages every 3 days after the initiation of subculture. A medium, in which 10% fetal bovine serum (FBS; Thermo Fischer Scientific, Waltham, MA)-added RPMI 1640 Medium (Sigma-Aldrich, St. Louis, MO) had been added with the penicillin-streptomycin solution (× 100) (FUJIFILM Wako Pure Chemical Corporation, Osaka, Japan), was used for culture. On the day of subcutaneous inoculation, tumor cells were collected to be washed twice with PBS. C1498-mWT1, prepared to be 2.0 × 10^5^ cells/50 μL of PBS, was subcutaneously inoculated into the right dorsal region of mice. A vernier caliper applied to the skin surface of animals was used to percutaneously measure tumor volume—the product of length × width × height divided by 2—until the moment when the diameter exceeded 20 mm. The day of subcutaneous inoculation was considered as day 0, and successful engraftment (tumor volume: 10–50 mm^3^) was verified on day 7 before using animals in the present study. Body weight was also examined every week.

The oral administration of the vaccine by gavage to tumor-bearing mice was initiated on day 8. The following 3 study groups (*n* = 13 each) were formed: the *B. longum* 420 group (2.0 × 10^9^ CFUs/100 μL of PBS); the *B. longum* 2656 group (2.0 × 10^9^ CFUs/100 μL of PBS); and the *B. longum* 420/2656 combination group (4.0 × 10^9^ CFUs/200 μL of PBS).

Animals were orally given the vaccine (1 dose/day; 5 doses/week) and were eventually sacrificed by the intraperitoneal administration of pentobarbital (120 mg/kg). Tumor volume was measured every 3 days between days 7 and 16, and every 2 days between days 16 and 24. On day 20, the correlation between tumor volume and WT1-specific CTL frequency in CD8^+^ T cells in peripheral blood (PB) was examined.

### WT1-specific CTLs

Twenty-eight female C57BL/6 J (H-2D^b^) mice were used to form the following 4 study groups: the *B. longum* wild type group (2.0 × 10^9^ CFUs/100 μL of PBS, 5 doses/week; *n* = 7); the *B. longum* 420 group (2.0 × 10^9^ CFUs/100 μL of PBS, 5 doses/week; *n* = 7); the *B. longum* 2656 group (2.0 × 10^9^ CFUs/100 μL of PBS, 5 doses/week; *n* = 7); and the *B. longum* 420/2656 combination group (4.0 × 10^9^ CFUs/200 μL of PBS, 5 doses/week; *n* = 7). PB (100 μL) from the tail vein of mice was collected into 500 μL of PBS added with 1% heparin (Mochida Pharmaceutical, Shinjuku-ku, Tokyo, Japan). The resulting solution, added with 500 μL of the 2% dextran (Nacalai Tesque, Kyoto, Japan) solution before pipetting, was put into the heat block that was left stationary at 37 °C for 20 min to isolate leukocytes. The collected supernatant solution, 800 μL, was centrifuged at 2400 rpm to obtain peripheral blood mononuclear cells (PBMCs). Hemolysis was conducted for 3 min and was then discontinued with 3 mL of staining medium (SM): (2% FBS-added PBS). PBMCs were washed twice with 1000 μL of SM and were then suspended with 100 μL of SM. A human Fc receptor-blocking reagent, Clear Back® (2 μL; MEDICAL & BIOLOGICAL LABORATORIES (MBL), Minato-ku, Tokyo, Japan), was added. Cell surface markers for flow cytometry were conducted as described below. An H-2D^b^ anti-WT1 tetramer, RMFPNAPYL-PE (0.5 μL; MBL), was added. Subsequently, the following monoclonal antibodies (mAbs) were added: anti-mouse CD3 mAb FITC (0.5 μL; Clone 17A2; eBioscience, San Diego, CA); anti-mouse CD8 mAb Alexa Fluor® 647, clone KT15 (1 μL; MBL); anti-mouse CD44 mAb PE/Cyanine 7, clone IM7 (0.5 μL; BioLegend, San Diego, CA); CD62L anti-mouse mAb APC/Cyanine7, clone MEL-14 (0.5 μL, BioLegend). PBMCs were washed twice with SM and were then suspended in 200 μL of SM. A DNA-binding dye 7-AAD (2 μL; BD Biosciences, Franklin Lakes, NJ) was added to stain dead PBMCs. FACSCanto™ II (BD Biosciences, Piscataway, NJ) was used to conduct the flow cytometry of PBMCs. The data were then analyzed with the Flow Jo software (Tree Star, Ashland, OR).

### Intracellular cytokines

Twenty female C57BL/6 J (H-2D^b^) mice were used to form the following 5 study groups: 2 control groups—the PBS group (100 μL, 5 doses/week for 4 weeks; *n* = 4) and the *B. longum* wild type group (2.0 × 10^9^ CFUs/100 μL of PBS, 5 doses/week for 4 weeks; *n* = 4)—; the *B. longum* 420 group (2.0 × 10^9^ CFUs/100 μL of PBS, 5 doses/week for 4 weeks; *n* = 4); the *B. longum* 2656 group (2.0 × 10^9^ CFUs/100 μL of PBS, 5 doses/week for 4 weeks; *n* = 4); and the *B. longum* 420/2656 combination group (4.0 × 10^9^ CFUs/200 μL of PBS, 5 doses/week for 4 weeks; *n* = 4). Mice were sacrificed after the termination of the oral administration to remove the spleen, and splenocytes were cultured with WT1_35–52_ peptide 5 mM (Scrum, Koto-ku, Tokyo, Japan) in complete medium at 37 °C. The half of the medium was changed every 2 days after day 2 of culture. On day 2 of culture or later, recombinant interleukin (rIL)-2 (Shionogi Pharmaceutical, Chuo-ku, Osaka, Japan) 20 IU/mL was added. On day 12 of culture, 4-hour culture with 10% FBS RPMI was conducted by pulsing or not pulsing cells with WT1_35–52_ peptide 10 μM and by retaining intracellular interferon-gamma (IFN-γ) with brefeldin A (FUJIFILM Wako Pure Chemical, Chuo-ku, Osaka, Japan) 10 μg/mL. These cultured cells were washed twice with SM before suspension in 50 μL of SM. Clear Back® (2 μL; MBL) was added. Subsequently, the following mAbs were added: anti-mouse CD3 mAb FITC, clone 17A2 (0.5 μL; eBioscience); and anti-CD4 mAb APC/Cyanine 7, clone RM4–5 (0.5 μL; BioLegend). Cells were stained at 4 °C for 30 min, followed by washing twice with SM. Subsequently, BD Cytofix/Cytoperm™ (BD Biosciences) was used to permeabilize cell membranes. Intracellular cytokine staining of IFN-γ was conducted with anti-IFN-γ mAb PE/Cyanine 7, clone XMG 1.2 (0.5 μL; BioLegend) at 4 °C for 30 min. The flow cytometry of splenocytes and the analysis of the resulting flow cytometric results were conducted as mentioned above.

### Tumor-infiltrating lymphocytes (TILs)

Eight female CD45.1^+^ C57BL/6 J (H-2D^b^) mice were used to form the following 2 study groups: the *B. longum* 420 group (2.0 × 10^9^ CFUs/100 μL of PBS, 5 doses/week for 2 weeks; *n* = 4); and the *B. longum* 420/2656 combination group (4.0 × 10^9^ CFUs/200 μL of PBS, 5 doses/week for 2 weeks; *n* = 4). C1498-mWT1, prepared to be 2.0 × 10^5^ cells/50 μL of PBS, was subcutaneously inoculated into the right dorsal region of mice. The day of subcutaneous inoculation was considered as day 0, successful engraftment was verified on day 7, and the oral administration of the vaccine was initiated on day 8. After the termination of the oral administration, the tumor mass was cut into small pieces that were then treated with enzymes of Tissue Dissociation Kit (Milteny Biotec, Bergisch-Gladbach, Germany), followed by mechanical dissociation with gentleMACS™ Dissociator (Milteny Biotec). The resulting cell suspension was strained with the 40-μm nylon cell strainer (Nippon Rikagaku Kikai, Bunkyo-ku, Tokyo, Japan) to obtain a cell population containing tumor cells and hemocytes.

WT1-specific CTLs were stained as described below. MojoSort™ (BioLegend, San Diego, CA), a magnetic cell separation system, was used to conduct the positive selection of CD45.1^+^ cells in an attempt to exclude tumor cells—CD45.2^+^ cells. Sorted CD45.1^+^ cells were then suspended in 200 μL of SM. Clear Back® (4 μL; MBL) was added in the resulting cell suspension. An H-2D^b^ anti-WT1 tetramer, RMFPNAPYL-PE (1.0 μL; MBL), was then added. Subsequently, the following mAbs were added: anti-mouse CD3 mAb FITC, clone 17A2 (2.0 μL; eBioscience); anti-mouse CD8 mAb Alexa Fluor® 647, clone KT15 (2.0 μL; MBL); and anti-mouse CD45.2 mAb PerCP/Cyanine 5.5, clone 104 (1.0 μL; BioLegend). TILs were washed twice with SM. A DNA-binding dye 7-AAD (10.0 μL; BD Biosciences) was added to stain dead TILs. The flow cytometry of TILs and the analysis of the flow cytometric results were conducted as mentioned above.

Intracellular cytokines of CD4^+^ T cells were stained according to the following two procedures: 1) CD4^+^ T cells were positively selected with Mojo Sort™ Mouse CD4^+^ T cell isolation Kit (BioLegend), followed by 2-week co-culture with feeder cells previously irradiated (40 Gy) to prevent cellular proliferation—CD45.2^+^ spleen cells (CD4^+^ T cell-spleen cell ratio—1:2) in the complete medium containing rIL-2 (Shionogi Pharmaceutical) 20 IU/mL and WT1_35–52_ peptide 20 μg/mL; and 2) After 2-week co-culture, IFN-γ was stained as mentioned above.

### Statistical analysis

Two-tailed Student’s t test was conducted for between-group comparisons, and Wilcoxon signed rank test for intergroup comparisons. Pearson’s product-moment correlation coefficient was determined to examine the correlation between tumor volume and WT1-specific CTL frequency in PB. A *p* value of < 0.05 was considered statistically significant. JMP® version 16.0.0 (SAS Institute Inc., Cary, NC) and the SAS software version 9.4 (SAS Institute Inc.) were used for all statistical analyses.

## Results

**Fig. 1:**
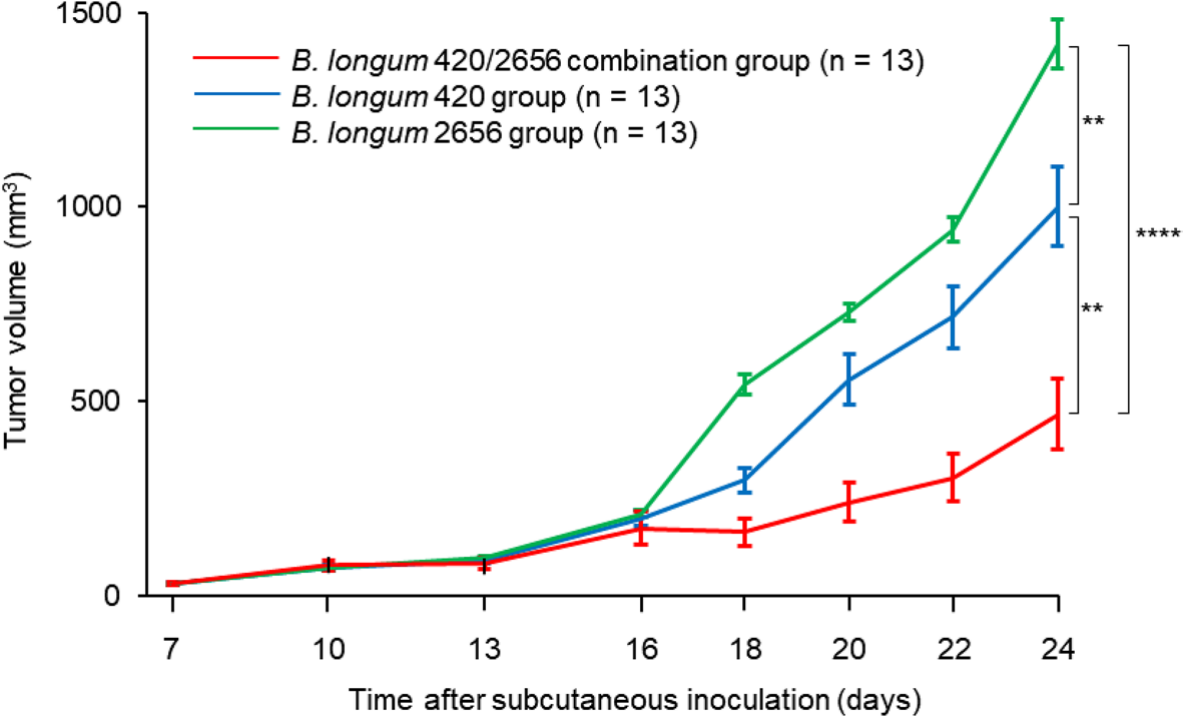
Time-course changes in tumor volume in the *B. longum* 420, 2656, and 420/2656 combination groups. Values are expressed as mean ± SE. **: *p* < 0.01; ****: *p* < 0.0001

**Fig. 2:**
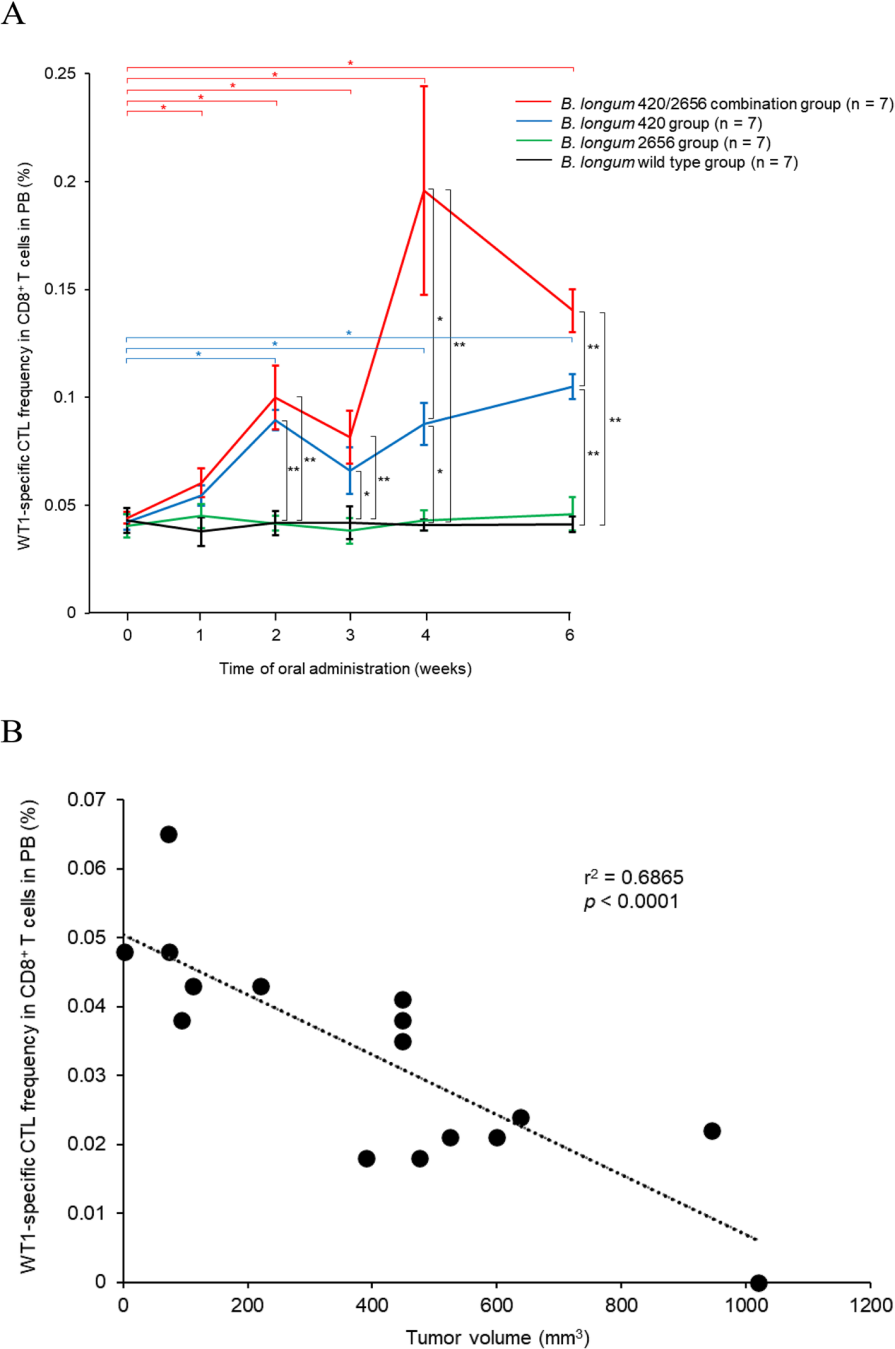
**A** Time-course changes in WT1-specific CTL frequency in CD8^+^ T cells in PB. WT1, Wilms’ tumor 1; CTL, cytotoxic T lymphocyte; PB, peripheral blood. Values are expressed as mean ± SE. *: *p* < 0.05; **: *p* < 0.01. **B** Correlations between WT1-specific CTL frequency in CD8^+^ T cells in PB and tumor volume on day 20 after subcutaneous inoculation to mice in the *B. longum* 420 and *B. longum* 420/2656 combination groups. WT1, Wilms’ tumor 1; CTL, cytotoxic T lymphocyte; PB, peripheral blood

**Fig. 3:**
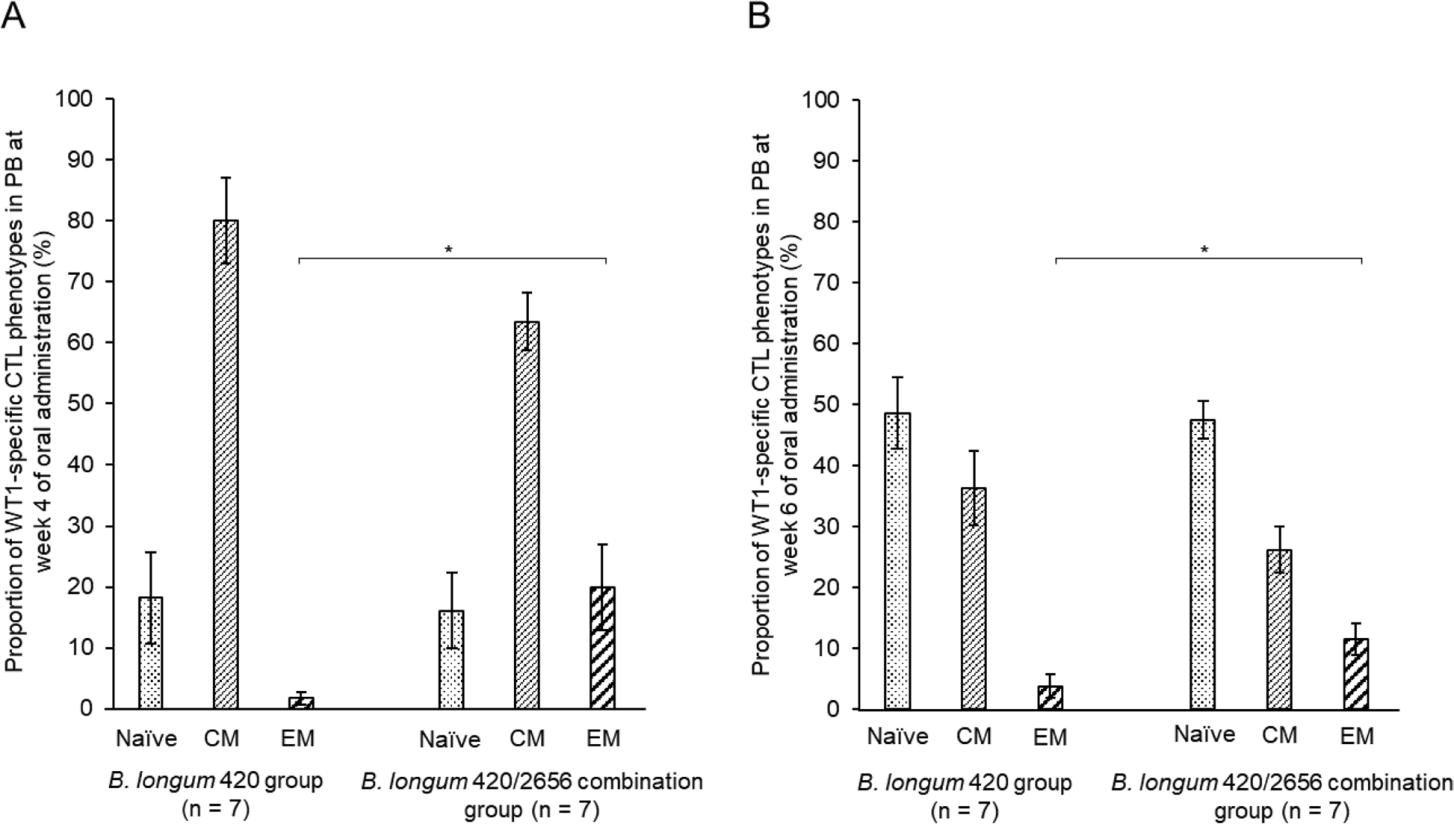
Proportions of WT1-specific CTL phenotypes in PB at weeks 4 (**A**) and 6 (**B**) of oral administration. Values are expressed as mean ± SE. *: *p* < 0.05. WT1, Wilms’ tumor 1; CTL, cytotoxic T lymphocyte; PB, peripheral blood

Tumor volume was significantly smaller in the *B. longum* 420/2656 combination group than in the *B. longum* 420 group and the *B. longum* 2656 group (Fig. 1; *p* < 0.01 and *p* < 0.0001, respectively) and was also significantly smaller in the *B. longum* 420 group than in the *B. longum* 2656 group (Fig. 1; *p* < 0.01) on day 24 after the subcutaneous inoculation of C1498-mWT1. No weight loss was found in all groups. WT1-specific CTL frequency in CD8^+^ T cells in PB was significantly greater in the *B. longum* 420/2656 group than in the *B. longum* 420 group at week 4 of oral administration (*p* < 0.05) and lasted up to week 6 (*p* < 0.01) (Fig. 2A). Moreover, the frequency increased significantly (*p* < 0.05) from the baseline values in the *B. longum* 420/2656 group since week 1 and in the *B. longum* 420 group at weeks 2, 4, and 6 (*p* < 0.05 each). In contrast, the frequency remained unchanged in the *B. longum* 2656 and *B. longum* wild type groups throughout the study period (Fig. 2A). On day 20 after subcutaneous inoculation, WT1-specific CTL frequency in CD8^+^ T cells in PB was inversely correlated with tumor volume (Fig. 2B; r^2^ = 0.6865, *p* < 0.0001) in mice treated with *B. longum* expressing WT1-derived CTL epitope including amino acid sequences 126–134 RMFPNAPYL in the *B. longum* 420 and *B. longum* 420/2626 combination groups. The proportion of the WT1-specific, effector memory (EM) CTLs in PB increased significantly in the *B. longum* 420/2656 combination group than in the *B. longum* 420 group at weeks 4 (Fig. 3A) and 6 (Fig. 3B) of oral administration (*p* < 0.05 each).

**Fig. 4:**
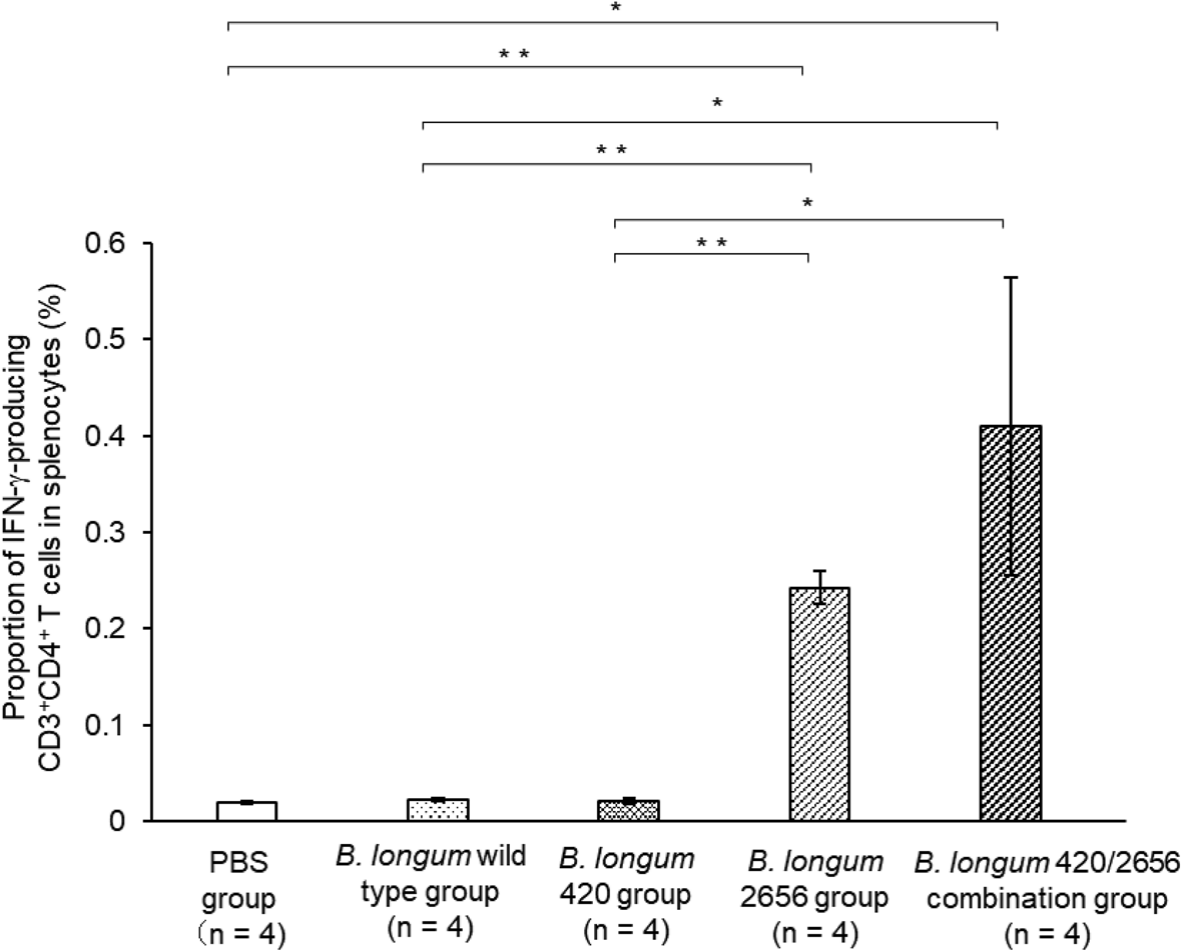
Proportions of IFN-γ-producing CD3^+^CD4^+^ T cells in splenocytes. Values are expressed as mean ± SE. *: *p* < 0.05; **: *p* < 0.01. IFN-γ, interferon-gamma

**Fig. 5:**
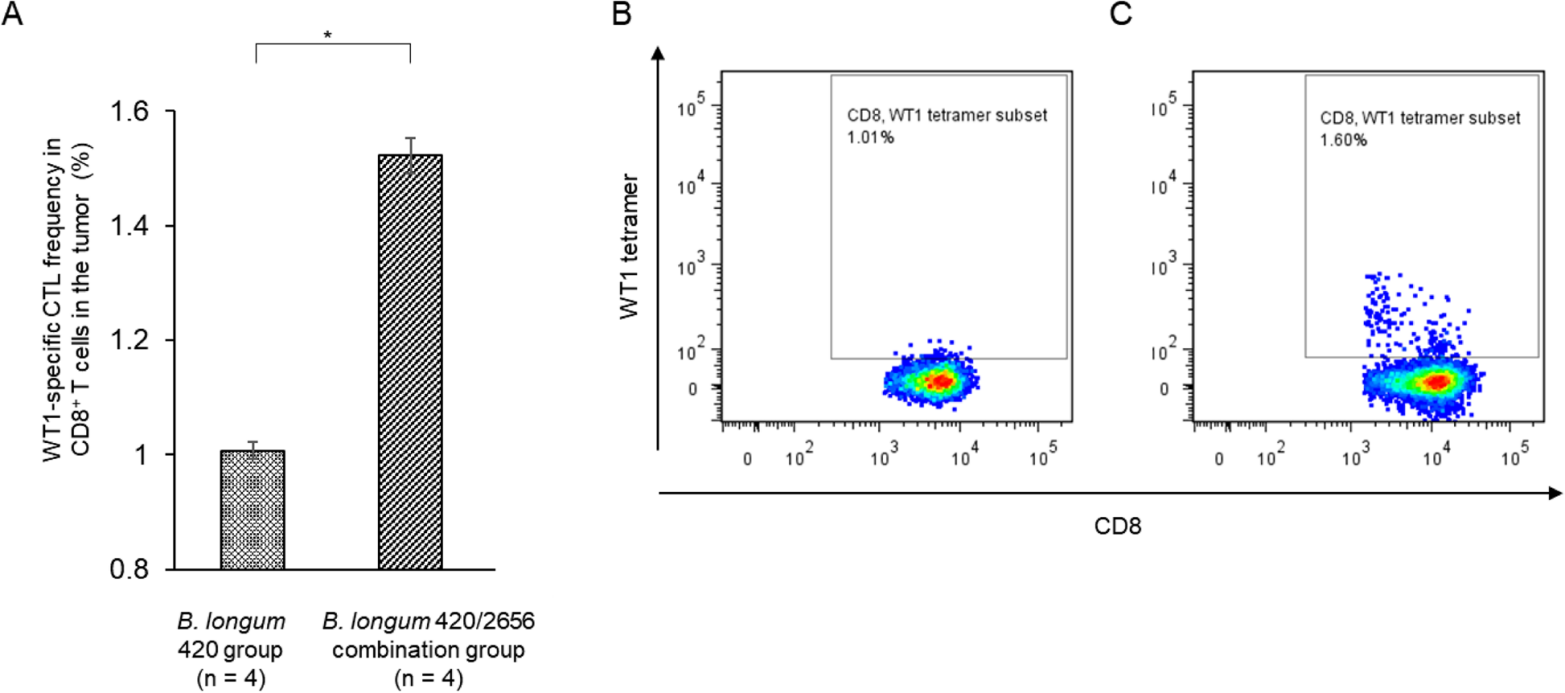
**A** WT1-specific CTL frequency in the tumor. Representative flow cytograms indicating WT1-specific CD3^+^CD8^+^ T cells in TILs (**B**). *B. longum* 420 group and (**C**). *B. longum* 420/2656 combination group. Values are expressed as mean ± SE. *: *p* < 0.05. CTL, cytotoxic lymphocyte; WT1, Wilms’ tumor 1; TIL, tumor-infiltrating lymphocyte

**Fig. 6:**
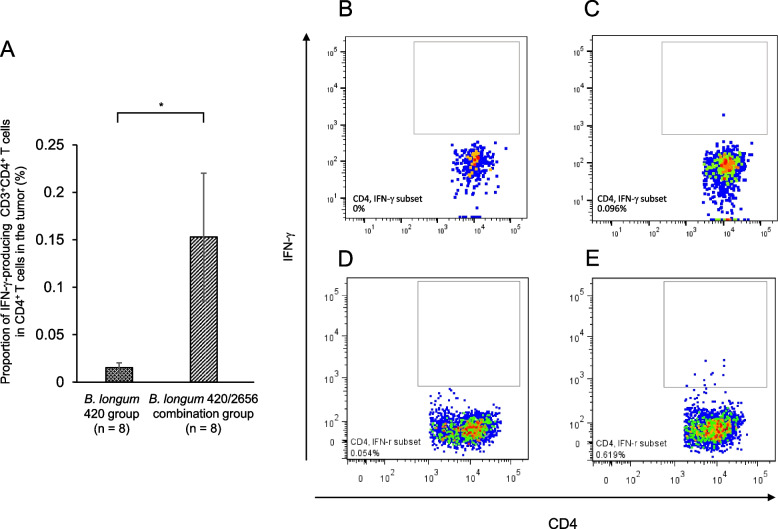
**A**. Proportion of IFN-γ-producing CD3^+^CD4^+^ T cells in CD4^+^ lymphocytes in the tumor. Representative flow cytograms indicating IFN-γ-producing CD3^+^CD4^+^ T cells in CD4^+^ T cells in the tumor not pulsed (**B**) or pulsed (**C**) with WT1_35–52_ peptide in the *B. longum* 420 group and not pulsed (**D**) or pulsed (**E**) with WT1_35–52_ peptide in the *B. longum* 420/2656 group. Values are expressed as mean ± SE. *: *p* < 0.05. IFN-γ, interferon-gamma

The proportion of WT1_35–52_ peptide-specific IFN-γ-producing CD3^+^CD4^+^ T cells in splenocytes was low in the PBS, *B. longum* wild type, and *B. longum* 420 groups. On the other hand, the proportion was significantly greater in the *B. longum* 2656 group (*p* < 0.01) and the 420/2656 combination group (*p* < 0.05) than in the *B. longum* wild type group, and was significantly greater (*p* < 0.05) in the *B. longum* 420/2656 combination group than in the 420 group (Fig. 4); no statistically significant difference (*p* = 0.11) was found in the proportion of IFN-γ-producing CD3^+^CD4^+^ T cells in splenocytes between these two groups. WT1-specific CTL frequency in TILs was significantly greater (*p* < 0.05) in the *B. longum* 420/2656 combination group than in the *B. longum* 420 group (Fig. 5A), with representative flow cytograms (Figs. 5B—420 group, C—420/2656 group). The proportion of IFN-γ-producing CD3^+^CD4^+^ T cells in CD4^+^ T lymphocytes in tumors was significantly greater (*p* < 0.05) in the *B. longum* 420/2656 combination group than in the *B. longum* 420 group (Fig. 6A), with representative cytograms (Figs. 6B, C—420 group, D, E—420/2656 group). The frequency of WT1_35–52_ peptide-specific INF-γ-producing CD3^+^CD4^+^ T cells in TILs of mice was scarcely detected in the *B. longum* 420 group (Figs. 6B, C) but was sufficiently detected in *B. longum* 420/2656 combination group (Figs. 6D, E).

## Discussion

Nakajima et al. reported that MHC class II-restricted, WT1 protein-derived helper epitopes—WT1_35_, WT1_86_, and WT1_294_ for WT1-speicific CD4^+^ T cells—play an essential role in WT1-specific tumor immunity in in vivo mouse models [15]. Among these, we selected WT1_35_ as an epitope with the most potent IFN-γ-producing capability of CD4^+^ T cells that strengthen the CTL-induced WT1-specific lysis of RMAS cells pulsed with WT1_35–52_ peptide, suggesting its great potential of inducing CD4^+^ T cell help that provides critical cellular and molecular mechanisms in CTL responses in cancer immunotherapy [12]. In addition, Nakagawa et al. demonstrated that an oral WT1 protein vaccine of WT1-anchored, genetically engineered *B. longum* provokes intestinal immunity to exert the cytotoxic activity of WT1-specific CD8^+^ T cells possibly through CD4^+^ T cell help [16]. Results from the present study indicate that the combination of *B. longum* 420 and *B. longum* 2656 exhibited the most enhanced anticancer activity in intestinal immunity among studied materials, supporting the reasonable therapeutic strategy of genetically engineering a WT1 amino acid sequence 35–52 into an oral *B. longum*-based vaccine structure.

Recent technologies including flow cytometry and single-cell RNA sequencing provide an unprecedented view of the composition, function, and location of immunocompetent cells within the tumor microenvironment (TME) [17]. Our data on TILs indicate 1) the increased frequency of WT1-specific CTLs in CD8^+^ T cells, 2) the obvious production of WT1-specific IFN-γ by CD4^+^ T cells, and 3) the strengthened tumor-infiltrating capability of WT1-specific CTLs primed in the intestine [16], all of which afford flow cytometric evidence about the antitumor activity of combining *B. longum* 420 for WT1-specific CD8^+^ T cell activation and *B. longum* 2656 for CD4^+^ T cell help.

In recent years, the TME—the site where immune cells of innate immunity (natural killer cells, neutrophils, and macrophages) harbor—has become one of research topics in developing more efficient immune therapies because of the following facts: antigen-presenting cells represented by dendritic cells (DCs) capture and cross-present tumor antigens released by tumor cells, and activate T cells [17, 18]; and immunosuppression entailing immune evasion [19] occurs that leads to tumor resistance [20, 21], tumor progression [22], tumor metastasis [17], and T cell depletion [23].

IFN-γ, when released from activated T cells, mediates the prevention of cancer cell proliferation [24], supports the effector responses of CD8^+^ CTLs, and is a surrogate marker for the detection of antigen-specific T cells [25]. Yokota et al. described that WT1-specific CTL frequencies in PB and tumors were correlated [26]. Additionally, our data indicate that 1) WT1-specific CTL frequency was significantly greater (*p* < 0.05) in the *B. longum* 420/2656 combination group than in the *B. longum* 420 group and 2) WT1-specific CTL frequency in CD8^+^ T cells in PB was inversely correlated with tumor volume. Taken together, the further acceleration of the antitumor activity of *B. longum* 420/2656 combination that relies on the IFN-γ-producing ability of cytotoxic effector cells [27] whose proportion in the tumor presumably increased.

Ahrends et al. described that CD4^+^ T cell help is essential for CTLs when exerting their cytotoxic activity through the downregulation of coinhibitory receptors, as well as increased motility and migration capacities [28]. CD4^+^ T cell help improves the clonal expansion of CTLs and their differentiation into effector and memory CTLs [12]. *B. longum* 420/2656 combination significantly increased the production of EM CTLs from naïve T cells at week 4 of oral administration, lasting up to week 6. Namely, we consider that the combination appreciably contributed to the sustainment of the antitumor activity of this vaccine.

Our previous study [16], in which mice were orally treated *B. longum* wild type, *B. longum* wild type displaying only GLBP, and *B. longum* 420, provided insights into a novel, intestinal bacterium-based, cancer immunotherapy through intestinal immunity and afforded the following findings: 1) CD8^+^ T cells played a central role in the cytotoxic activity of *B. longum* 420; 2) only *B. longum* 420 augmented the efficiency of T cell priming; and 3) CD4^+^ T cell help was evidenced by the production of anti-WT1 IgG antibody and by the increased number and proportion of IFN-γ-producing CD4^+^ T cells in the Peyer’s patches (PPs) and mesenteric lymph nodes. Intestinal immunity is an attractive immune mechanism in intestinal bacterium-based, cancer immunotherapy because an oral peptide/protein vaccine stimulates lamina propria and PP immune cells (e.g., DCs, T- and B-cells, and macrophages) and these stimulated immune cells may reach systemic circulation through lymphocyte network—the site where the efficient recognition and presentation of a TAA by DCs occur. Namely, the development of an intestinal bacterium-based, oral WT1 protein vaccine containing a helper epitope is therapeutically highly reasonable based on the following facts: 1) more potent antigenicity is endowed by *B. longum* that acts as an adjuvant and presumably by WT1 protein (a substance of greater molecular size than WT1 peptide) that generates a larger number of epitopes when processed by DCs; 2) WT1_35_ helper epitope helps the production of CTLs in the intestinal immune system, thus enhancing the cytotoxic activity of a cancer vaccine; and 3) WT1-stimulated immune cells may reach systemic circulation more extensively via the intestinal than dermal immune system, with a greater likelihood of activating and sustaining immune responses—natural or acquired. Our data concerning the increased frequency of WT1-specific CTLs in CD8^+^ T cells in PB and the elevated proportion of WT1-specific IFN-γ-producing CD3^+^CD4^+^ T cells in splenocytes indicate the activation of immune cells and the sustainment thereof in peripheral circulation and lymphoid tissue.

*B. longum* 420, alone or in combination with *B. longum* 2656, caused no weight loss. *B. longum* 420/2656 combination is inexpensive, is easy to administer, and acquires good patient adherence based on the fact that *B. longum* is a safety-recognized probiotic. However, the safety and efficacy of *B. longum* 420/2656 combination need to be examined in clinical trials enrolling cancer patients, especially pediatric and elderly patients. We consider that *B. longum* 420/2656 combination following treatment with other anticancer agents for adult cancer patients (e.g., immune checkpoint inhibitors and anticancer chemotherapeutic agents) or immunotherapies for pediatric cancer patients (e.g., allogeneic hematopoietic stem cell transplantation) would intensify the anticancer efficacy of the relevant therapeutic modalities.

In the present study, we did not examine intestinal immunity through immunohistochemistry and immunostaining because our previous study of *B. longum* 420 [16] had indicated that the PPs represent the anatomic site where immune responses are triggered by *B. longum* 420. We could not investigate the influences of *B. longum* 420/2656 combination on the activation of DCs in the PPs. The *B. longum* 2656-to-420 ratio (1:1), which was established based on their CFUs, needs to be investigated in more depth. State-of-the-art technologies including single-cell RNA sequencing of immune cells will clarify whether differences in immune responses induced by traditional and oral vaccines are due to differences in the anatomophysiological features of immune cells (e.g., phenotype pattern, source of origin, and degree of maturation).

## Conclusion

We examined the antitumor activity of an intestinal bacterium-based, oral WT1 protein vaccine in a model of murine leukemia, and *B. longum* 420/2656 combination further accelerated antitumor activity that relies on WT1-specific CTLs in the tumor compared with *B. longum* 420. The vaccine successfully triggered and sustained CD4^+^ T cell help-enhanced immune responses in the intestinal tract, and then in systemic circulation and the spleen, thus allowing WT1-specific T cells to reach the tumor where they exerted obvious antitumor activity.

## Data Availability

The datasets analyzed during the current study are available from the corresponding author on a reasonable request.

## Abbreviations

*B. longum*: *Bifidobacterium longum*
C1498-mWT1: C1498-murine WT1
CFU: Colony-forming unit
CTL: Cytotoxic T lymphocyte
DC: Dendritic cell
FBS: Fetal bovine serum
EM: Effector memory
GAM: GIFU Anaerobic Medium
GLBP: Galacto-N-biose/lacto-N-biose I-binding protein
HLA: Human leukocyte antigen
mAb: Monoclonal antibody
IFN-γ: Interferon-gamma
MHC: Major histocompatibility complex
PB: Peripheral blood
PBMC: Peripheral blood mononuclear cell
PBS: Phosphate-buffered saline
PP: Peyer’s patch
rIL: Recombinant interleukin
SM: Staining medium
TAA: Tumor-associated antigen
TIL: Tumor-infiltrating lymphocyte
TME: Tumor microenvironment
WT1: Wilms’ tumor 1
